# Clinical performance and exercise hemodynamics in patients with severe secondary tricuspid regurgitation and chronic atrial fibrillation

**DOI:** 10.1186/s12872-021-02094-3

**Published:** 2021-06-04

**Authors:** Jesper K. Jensen, Tor S. Clemmensen, Christian A. Frederiksen, Joachim Schofer, Mads J. Andersen, Steen H. Poulsen

**Affiliations:** 1 Department of Cardiology, Aarhus University Hospital, Aarhus, 8200 Denmark; 2 MVZ Department Structural Heart Disease, Asklepios Clinic St.Georg, Hamburg, Germany

**Keywords:** Atrial fibrillation, Secondary tricuspid regurgitation, Cardiac amyloid cardiomyopathy, Exercise hemodynamics

## Abstract

**Objective:**

The study aimed to investigate the functional capacity and hemodynamics at rest and during exercise in patients with chronic atrial fibrillation and severe functional symptomatic tricuspid regurgitation (AF-FTR).

**Background:**

Symptoms and clinical performance of severe AF-FTR mimic the population of patients with heart failure with preserved ejection fraction (HFpEF). Severe AF-FTR is known to be associated with an adverse prognosis whereas less is reported about the clinical performance including exercise capacity and hemodynamics in patients symptomatic AF-FTR.

**Methods:**

Right heart catheterization (RHC) at rest and during exercise was conducted in a group of patients with stable chronic AF-TR and compared with a group of patients with HFpEF diagnosed with cardiac amyloid cardiomyopathy (CA). All patients had preserved ejection fraction and no significant left-sided disease.

**Results:**

Patients with AF-FTR demonstrated a low exercise capacity that was comparable to CA patients (TR 4.9 ± 1.2 METS vs. CA 4. 7 ± 1.5 METS; *P* = 0.78) with an average peak maximal oxygen consumption of 15 mL/min/kg. Right atrium pressure increased significantly more in the AF-FTR patients as compared to CA patients at peak exercise (25 ± 8 vs 19 ± 9, p < 0.01) whereas PCWP increased significantly to a similar extent in both groups (31 ± 4 vs 31 ± 8 mmHg, p = 0.88). Cardiac output (CO) was significantly lower among AF-FTR at rest as compared to CA patients (3.6 ± 0.9 vs 4.4 ± 1.3 l/min; p < 0.05) whereas both groups demonstrated a poor but comparable CO reserve at peak exercise (7.3 ± 2.9 vs 7.9 ± 3.8 l/min, p = 0.59).

**Conclusions:**

AF-FTR contributes to the development of advanced heart failure symptoms and poor exercise capacity reflected in increased atrial filling pressures, reduced cardiac output at rest and during exercise sharing common features seen in HFpEF patients with other etiologies.

## Introduction

Among patients diagnosed as heart failure with preserved left ventricular ejection fraction (HFpEF), atrial fibrillation (AF) is a common finding at presentation and AF is closely associated to known etiologies of HFpEF as hypertension, diabetes mellitus and ageing [1–3]. Therefore, AF is an important confounder of the diagnosis of HFpEF as well as to the diagnostic criteria used to define HFpEF [4]. Recently, it has been demonstrated that HFpEF patients with AF differs hemodynamically from those with sinus rhythm having higher pulmonary capillary wedge pressure (PCWP), mean pulmonary artery (PA) pressure and poorer exercise capacity [5]. Chronic or long-standing AF might in some patients lead to significant structural and functional changes with right and left atrial dilatation along with functional mitral or tricuspid valve insufficiency [6, 7]. Isolated functional tricuspid regurgitation (FTR) following chronic AF (AF-FTR) is characterized by tricuspid annular dilation and severe right atrial enlargement and accounts for 9% of patients with FTR and is associated with age, female gender and preserved left ventricular ejection fraction (LVEF) [7, 8]. From a clinical aspect these patients resemble patients categorized as heart failure with preserved LVEF (HFpEF) as they present with typical signs of clinical heart failure, enlarged atria, elevated natriuretic peptides and normal LVEF [9]. However, there is limited clinical and hemodynamics data available in patients with AF-FTR. A better understanding of these patients is wanted due to adverse prognosis and the fact that most patients will only receive diuretic medical therapy until intractable severe right heart failure appear [10]. As off today isolated tricuspid valve surgery remains relatively rare and the reluctance to perform isolated tricuspid valve (TV) surgery is likely to reflect the reported increased in-hospital mortality [11]. In recent years, interest in TV pathology have rapidly expanded in response to reported poor clinical outcome and as a consequence of emerging percutaneous transcatheter treatment options in untreated high-risk patients with severe FTR.[12] However, less focus has been on the contribution of severe AF-FTR on clinical performance including exercise capacity and hemodynamics. The importance of recognizing significant AF-FTR as a heart failure associated condition is crucial since the prevalence of this type of patients is likely to increase significantly in the future due to the expected increase of AF and HFpEF in the general aging population.

The present study aims to investigate the clinical heart failure characteristics, functional capacity and hemodynamics at rest and during exercise in patients with severe AF-FTR and compare these patients with patients with another HFpEF like condition as cardiac amyloid cardiomyopathy (CA).

## Methods

### Patients

A total of 22 consecutive clinical stable patients with chronic AF (> 2 years) and severe secondary TR were enrolled between November 2017 and October 2019 at the Department of Cardiology, Aarhus University Hospital, Denmark. All patients were referred to the out-patient clinic due to heart failure symptoms for evaluation. Patients with moderate to severe left-sided valve disease, LVEF < 50%, pacemaker devices and pulmonary disease were excluded. These patients were compared with 22 patients with restrictive cardiomyopathy due to confirmed CA (determined by endomyocardial biopsy using Congo red dye and immunohistochemistry) as follows: 10 patients with wild-type transthyretin, 4 patients with familial amyloidosis mutation carriers, and 8 patients with light-chain amyloidosis [13]. All control subjects (CA patients) had a LVEF ≥ 50% and no significant TR, 17 patients were in sinus rhythm, 5 patients were in AF, but did not differ with respect to LV mass, chamber size or LVEF. All patients with CA were without significant left-sided heart valve disease or significant TR at the time of the investigation.

### Ethics

All patients were aged ≥ 18 years and provided written informed consent according to the principles of the Helsinki Declaration. The local scientific ethical committee of the Central Denmark Region approved the study (1-10-72-399-17).

### Invasive hemodynamic assessment

Right heart catheterization (RHC) was conducted in all patients with a standard 7.5-F triple lumen Swan-Ganz thermistor and balloon-tipped catheter (Edwards Lifesciences, Irvine, CA, USA). RHC was performed through an 8-french sheath in the right internal jugular vein and advanced to the PA. The position of the catheter was assessed by the distinct pressure waveforms and fluoroscopy. At rest and at each level of exercise level until exhaustion, systolic, diastolic, mean pulmonary artery pressure (sPAP, dPAP, mPAP), mean right atrial pressure (RAP), PCWP, cardiac output (CO), were measured. Oxygen consumption (VO_2_) was measured using breath-by-breath expired gas analysis (Jaeger Master Screen CPX, CareFusion, 234 GmbH, Hoechberg, Germany). Arterial-venous O_2_ difference (A-VO_2_diff) was measured directly as the difference between systemic and PA O_2_ contents (O_2_ saturation × hemoglobin × 1.34). CO was calculated by direct Fick (CO = VO_2_/A-VO_2_diff). Stroke volume (SV) was calculated as CO/heart rate (HR) and indexed according to body surface area (BSA) as cardiac index (CI). Pulmonary vascular resistance (PVR) was calculated as: PVR = (mPAP-PCWP)/CO [14–16]. RV stroke work index (RWSVI) was calculated as: stroke volume index (SVI) × 0.0136 ×  (mPAP–mRAP) and left ventricular stroke work index as: SVI × 0.0136 ×  (mean blood pressure – mPCWP). Left ventricular (LV) transmural pressure (LVTMP) reflecting LV preload was calculated as PCWP-RAP [17, 18].

### Echocardiography

Transthoracic echocardiography was performed using the GE VIVID E95 system (GE Medical System, Horten, Norway) with a 3.5-MHz transducer for 2-D evaluation. We used the current guidelines for the assessment of chamber quantification [19, 20]. All measurements were averaged over 4–6 consecutive heart cycles in end-expiration. The following parameters were recorded by 2-dimensional echocardiography: LVEF, left ventricular end-diastolic (LVEDV) and systolic volume (LVESV) were assessed by the biplane disc method. Left and right atrial volume were assessed by biplane area-length method indexed by body surface area (BSA). The interventricular septum and the posterior wall thickness were obtained from the parasternal view. LV mass was calculated and indexed according to BSA. Right ventricular (RV) systolic function was assessed by the 3D-RVEF volumetric method, tricuspid annular systolic velocity (RV S´) measured by lateral, pulsed tissue Doppler velocities. In addition, tricuspid annular plane systolic excursion (TAPSE) was obtained from the lateral tricuspid plane. The tricuspid annulus was measured in a RV focused 4-chamber view and the severity of secondary tricuspid regurgitation was categorized as mild, moderate or severe based on current guidelines [20, 21]. EchoPAC version 203 (GE-Vingmed Ultrasound, Horten, Norway) was used for image analysis.

### Exercise protocol

Symptom-limited exercise was performed on a semi-supine bicycle (GE eBike L Ergometer, Freiburg, Germany). Work rate started at 0 W and was increased by 25 W every 3 min. The level of workload was determined by each patient based on self-reporting. The patients were encouraged to maintain a fixed pedalling speed of 60–65 rounds per minute and to exercise until reaching Borg scale 20 [22]. During the test, continuous 12-lead electrocardiogram and pulse oximetry were measured together with blood pressure. In order to determine oxygen uptake (VO_2_), gas exchange was measured using a breath-by-breath technique (Jaeger MasterScreen CPX, CareFusion, 234 GmbH, Hoechberg, Germany), Peak VO_2_ was defined as the averaged value during the final 15 s of exercise. The gas analyser was calibrated before each test.

### Statistical methods

Data are reported as means ± SD for normally distributed or median (interquartile range) for non-normally distributed variables. Categorical data is presented as absolute values with percentages. Between-group differences were tested using the 2-sample independent *t*-test, the Mann–Whitney U-test for non-normally distributed data and χ^2^ test for dichotomized data. We used linear regression models to compare continuous variables and predicted value and residual to check the models. All tests were two-sided and a *P* value < 0.05 was considered significant. Statistical analysis was performed using STATA/IC version 13.0 (STATACorp LP, College Station, TX, USA).

## Results

### Patients characteristics

The clinical characteristics of the patients are outlined in Table 1. All the patients were symptomatic (New York Heart Association functional classes II and III). The patients with AF-FTR were older, more frequently females and the prevalence of diabetes and hypertension was significantly higher as compared to CA patients. Both groups were comparable with respect to NT-proBNP, creatinine and medication, e.g. the use of betablockers and diuretics. The majority of AF-FTR patients had severe TR (91%) with a significantly enlarged TV annulus (47 ± 6 mm). Severe dilation of the left atrium was noted in both groups but without any significant difference between groups. As expected, wall thickness and LV mass were significantly increased in the CA group as compared to AF-FTR patients. RVEF and TAPSE were comparable and within normal range in both groups whereas RV S´ was higher in patients with AF-FTR compared with CA.

**Table 1:** Baseline characteristics

	TR patients (n = 22)	CA patients (n = 22)	P
Male (%)	50%	86%	0.01
BSA	2.0 ± 0.2	1.9 ± 0.2	0.59
Age (years)	79 [76;82]	71 [59;80]	< 0.05
Diabetes Mellitus (%)	18%	0%	< 0.05
Hypertension (%)	57%	23%	< 0.05
*Medication*			
Anticoagulants (%)	95%	23%	< 0.0001
ACE/ATII inhibitor (%)	45%	18%	0.05
Beta blockers (%)	55%	32%	0.13
Digoxin (%)	9%	0%	0.15
Loop diuretics (%)	77%	68%	0.50
Thiazide (%)	27%	9%	0.12
Spironolactone (%)	32%	18%	0.30
*Echocardiography* *Left ventricle*			
Ejection fraction (%)	59 [55;63]	58 [44;63]	0.62
End diastolic volume (mL/m2)	35 [27;47]	47 [43;56]	< 0.01
End systolic volume (mL/m2)	16 [13;22]	21 [17;29]	0.06
PWT (mm)	11 [10;12]	14.5 [13;17]	< 0.001
IVS (mm)	10 [10;12]	17 [13;22]	< 0.0001
Mass index (g/m2)	92 [77;106]	154 [123;203]	< 0.0001
Left atrial volume (mL/m2)	56 ± 21	47 ± 18	0.13
*Right ventricle*			
TV annulus (mm)	47 ± 6		
TV ERO (cm^2^)	0.5 [0.4;0.8]		
TVR volume (mL)	48 [38;57]		
End diastolic volume (mL/m2)	30 [22;35]	27 [21;31]	0.47
End systolic volume (mL/m2)	15 [10;20]	12 [10;16]	0.24
Ejection fraction (%)	52 ± 10	53 ± 9	0.66
TAPSE (mm)	21 ± 4	18 ± 7	0.07
S’ (cm/s)	11.2 ± 1.7	8.9 ± 2.6	< 0.01
Right atrial volume (ml/m2) *Biochemistry*	75 [55;105]	36 [22;47]	< 0.0001
Lactate at peak exercise (mmol/L)	5.3 [3.5;7.2]	4.5 [2.9–6.2]	0.53
Creatinine (µmol/L)	94 [71;115]	101 [84;128]	0.14
Hemoglobin (mmol/L)	8.3 [8.1;9.0]	8.5 [8.0;9.1]	0.60
NT-ProBNP (ng/L)	1245 [817;1849]	2762 [976;4176]	0.10

Data are presented as absolute number and present or mean ± standard deviation or median and IQR

* = p < 0.05. ACE = Angiotensin-converting-enzyme, AT = angiotensin, BSA = Body surface area, NT-Pro-BNP = N-terminal natriuretic brain natriuretic peptide; NYHA = New York heart association, IQR = interquartile range, PWT = posterior wall thickness, IVS = interventricular septum, LV = left ventricle, MV = mitral valve, S’ = tricuspid annular systolic velocity, TAPSE = tricuspid annular plane systolic excursion, TR = tricuspid valve, TV ERO = effective tricuspid valve regurgitant orifice; TVR = tricuspid valve regurgitant

### Invasive haemodynamic parameters at rest

The baseline resting haemodynamic characteristics of both groups are displayed in Table 2. The mean arterial blood pressure was significantly higher in patients with TR compared as to CA and CO was significantly lower among patients with TR (3.6 ± 0.9 vs. 4.4 ± 1.3 L/min; *P* < 0.05). The RAP was borderline significantly higher in patients with TR compared with CA (12 ± 5 mmHg vs. 9 ± 5 mmHg; *P* = 0.06). PCWP was slightly elevated in both groups at rest but without any significant differences between the AF-FTR patients and the CA group. The LV transmural pressure, RVSWI and LVSWI were comparable between groups at rest.

**Table 2:** Hemodynamic parameters at rest and at peak exercise

	Rest			Peak exercise		
	TR-patients (n = 22)	CA-patients (n = 22)	P	TR-patients (n = 21)	CA-patients (n = 22)	P
*Peak exercise (METS)*				4.9 ± 1.2	4.7 ± 1.5	0.78
*Hemodynamics*						
MAP (mmHg)	101 ± 16	90 ± 10	< 0.05	142 ± 37	106 ± 28	< 0.01
HR (beats/min)	74 ± 14	74 ± 13	0.92	131 ± 28	121 ± 26	0.23
AV-diff (%)	31 ± 5	34 ± 7	0.16	66 ± 12	72 ± 11	0.31
SVRI (dynes*s*cm^−5^*m^2^)	2999 [2434;4235]	2933 [2496–3485]	0.37	2362 [1859;3878]	1788 [1296;2281]	< 0.05
CO (L/min)	3.6 ± 0.9 ^#^	4.4 ± 1.3 ^¤^	< 0.05	7.3 ± 2.9 ^¤^	7.9 ± 3.7 ^¤^	0.59
CI (L/min/m^2^)	1.8 ± 0.4 ^#^	2.3 ± 0.6 ^¤^	< 0.01	3.7 ± 1.4 ^¤^	4.1 ± 1.7 ^¤^	0.48
VO_2_ (mL/min/kg)	4.0 ± 1.4	3.9 ± 0.7	0.86	15 ± 5	15 ± 5	0.71
mRAP (mmHg)	12 ± 5	9 ± 5	0.06	25 ± 8	16 ± 9	< 0.01
mPAP (mmHg)	28 ± 8	25 ± 9	0.20	49 ± 8	46 ± 12	0.42
PCWP (mmHg)	17 ± 5	14 ± 6	0.17	31 ± 4	31 ± 8	0.88
mRAP/mPCWP	0.8 [0.6;0.9]	0.6 [0.5;0.8]	0.13	0.8 [0.6;0.9]	0.5 [0.3;0.6]	< 0.001
LVTMP (mmHg)	4 [2;8]	4 [2;8]	0.50	6 [3;11]	17 [10;20]	< 0.01
PVRI (dynes*s*cm^−5^*m^2^)	428 [300;563]	261 [152;533]	0.22	457 [263;515]	272 [199;408]	0.11
PAC (mL/mmHg)	2.7 [1.9;3.2]	2.8 [4.2;2.1]	0.34	1.6 [1.1;1.9]	1.8 [1.6–2.7]	< 0.05
RVSWI (gm-m/m^2^/beat)	5.6 [4.5;8.1]	6.3 [4.1;8.7]	0.94	8.1 [5.5;10.3]	13.9 [7.9;21.0]	< 0.05
LVSWI (gm-m/m^2^/beat)	33 [27;43]	33 [24;40]	0.66	39 [26;61]	34 [18;52]	0.29

Data are presented as mean ± standard deviation

* = p < 0.05

MAP = mean arterial blood pressure; HR = heart rate; AV-diff = arterial-venous saturation difference; SVRI = systemic vascular resistance index, CI = cardiac index; VO2 = oxygen consumption; mRAP = mean right atrial pressure; mPAP = mean pulmonary arterial pressure; mPCWP = mean pulmonary capillary wedge pressure; LVTMP = left ventricular trans-mural pressure, PCWP = pulmonary capillary wedge pressure at PVRI = pulmonary vascular resistance index; PAC = pulmonary arterial compliance, RVSWI = right ventricular stroke work index, LVSWI = left ventricular stroke work index

### Invasive haemodynamic parameters during exercise

**Fig. 1:**
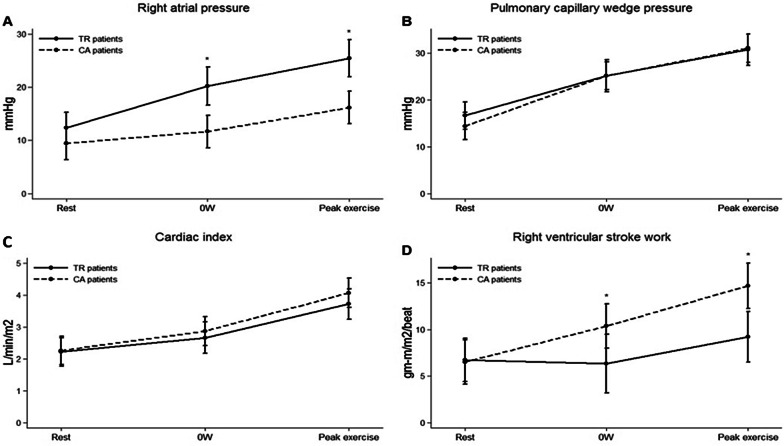
shows changes of mean right atrial pressure (**a**), pulmonary wedge pressure (**b**), cardiac index (**c**) and right ventricular stroke index (**d**) from rest, to exercise at 0 watts and at peak exercise. * denotes p < 0.01 between groups at difference exercise levels

As shown in Table 2, there was no significant difference in peak exercise capacity between patients with AF-FTR and CA and both groups demonstrated low exercise capacity (TR 4.9 ± 1.2 METS vs. CA 4.7 ± 1.5 METS; *P* = 0.78) with an average very low peak VO_2_ of 15 mL/min/kg. As shown in Fig. 1, RAP increased significantly more in the AF-FTR patients as compared to CA patients whereas PCWP increased significantly to the same extent in both groups. In addition, CI increased in both groups approximately only 2-folds whereas RVSWI was significantly lower in AF-FTR as compared to CA control subjects during exercise. Pulmonary venous hypertension was noted in both groups.

The ratio of changes from baseline to peak exercise for VO2 and CO (ΔVO2/ΔCO) was 0.35 [0.19;0.55] for AF-FTR patients which was comparable to the CA group [0.19;0.31]; p = 0.11. No significant correlations were found between maximal VO2 and atrial filling pressures or pulmonary artery pressures.

**Fig. 2:**
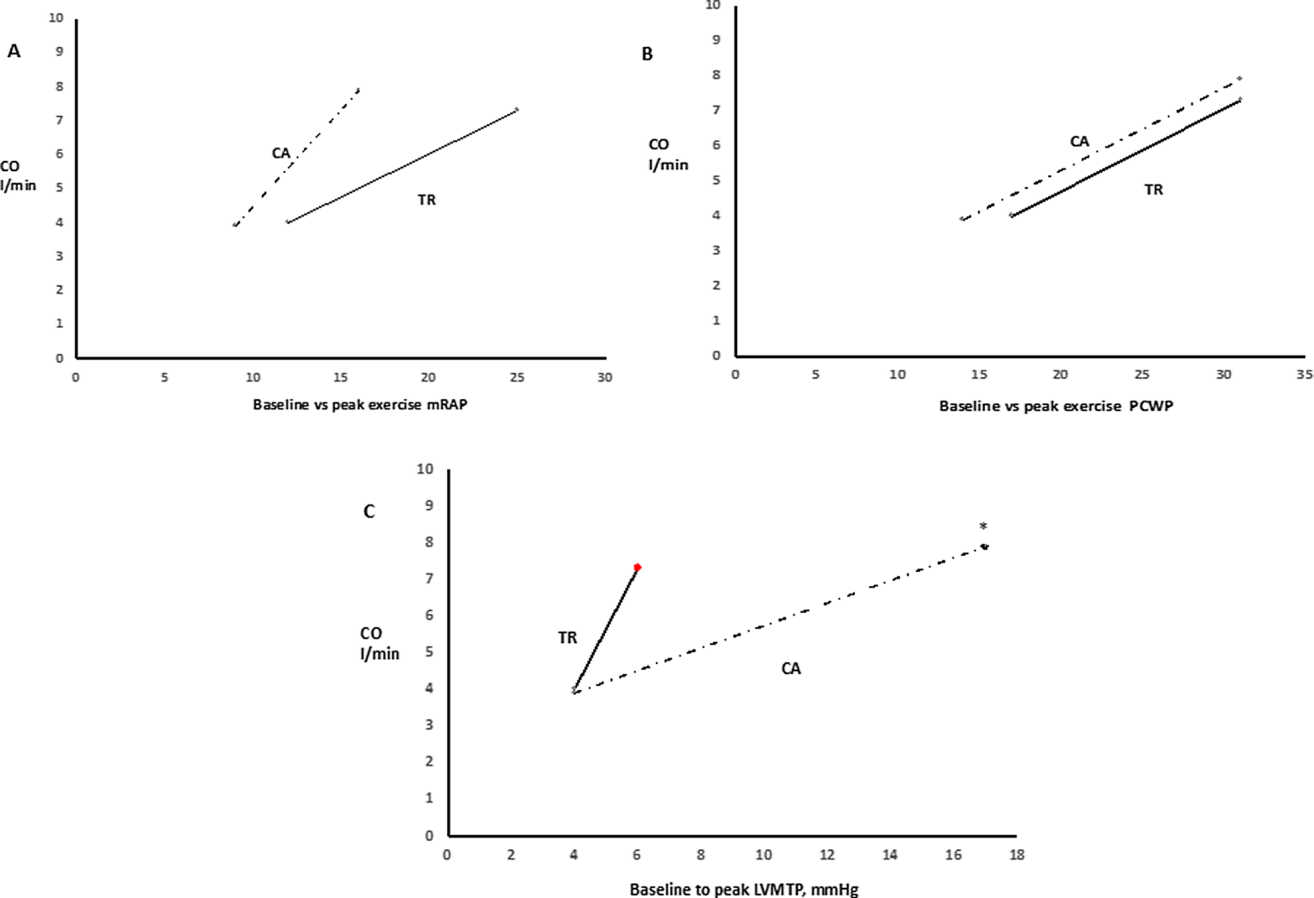
compares the changes of cardiac output in relation to changes in mean right atrial pressure (mRAP) (**a**), pulmonary wedge pressure (PCWP) (**b**), and left ventricular transmural pressure (LVTMP) (**c**) from rest to peak exercise in patients with AF-FTR and CA. * denotes p < 0.001 for comparison between the slopes of the two groups

The LV preload characteristics seems significantly less affected during exercise in AF-FTR as compared to CA control subjects as shown in Fig. 2. The response of CO to increased RV filling pressure during exercise seems reduced in AF-FTR compared to CA illustrated in Fig. 2.

**Fig. 3:**
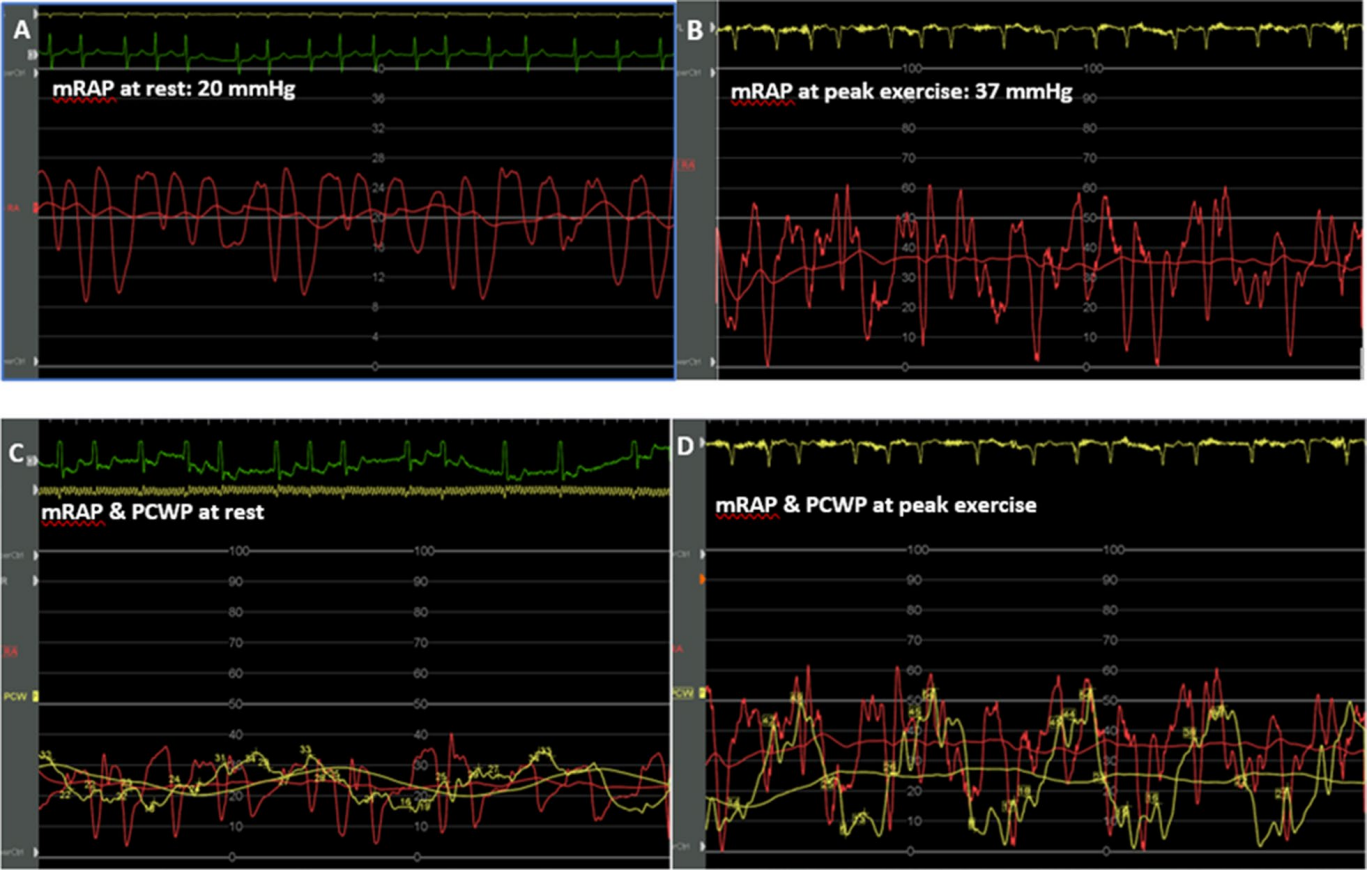
Typical right atrial pressure tracings at rest (**a**), note the large single regurgitant systolic wave and during peak exercise (**b**) in a patient with AF-FTR. Mean atrial pressure is significantly elevated at rest but raises even further with exercise. Of notice there is no drop in RA pressure during inspiration (Kussmaul sign) despite reduction in PCWP associated with decreasing intrathoracic pressure (C + D) which is especially pronounced at peak exercise

Typical changes of RAP and PCWP at rest and during exercise in a patient with AF-FTR are shown in Fig. 3, including simultaneous atrial pressure recordings demonstrating the Kussmaul sign.

## Discussion

This study examined the functional capacity and hemodynamic responses to exercise measured invasively in patients with chronic severe AF-FTR with preserved LVEF. For comparison, a group of patients with CA and HFpEF like condition were selected. The main findings are as follows: firstly, the patients with AF-FTR had advanced heart failure symptoms and demonstrated a low exercise capacity which was comparable to CA. Secondly, a reduced CO at rest was noted in addition to an abnormal cardiac output reserve response to stress in AF-FTR patients that was similar to the response seen in the CA group. Thirdly, in the AF-FTR group, severe RA enlargement was demonstrated with significantly increased filling pressures at rest as well during peak exercise. Of notice, the left-sided filling pressures were elevated at rest and particular during peak exercise in both patients with AF-FTR and CA. The LV preload characteristics evaluated by LVMTP differed significantly between the patient groups as the AF-FTR patients seemed to have preserved the LV preload properties in contrast to the expected and demonstrated restrictive LV preload properties in the CA patients. In contrast, the relation between RA filling pressures and CO during stress seems to be reduced in AF-FTR patients as compared to CA.

The risk of developing AF increases substantially with age and some will develop chronic AF. That is now increasingly accepted as a risk marker of FTR development [7, 23, 24]. The prevalence of AF in patients with moderate-severe FTR is reported as high as 9.2% and the presence of chronic AF is in particular related to the FTR severity [8, 24]. Chronic AF promotes in particular RA enlargement and TA dilation which increases the risk of development of FTR [25, 26]. The development of significant FTR in chronic AF seems not to be a valvular disease but rather an abnormality that is the result of disease processes that alters the TA, RA, RV size and function consequently leading to geometrical disturbance in the anatomical structures supporting the TV. Severe FTR among AF patients occur mostly in patients with chronic AF rather than paroxystic AF and a higher prevalence of right-sided heart failure has previous been reported in chronic AF which is in accordance with our findings.[8]. The presence of FTR may be considered as a mediator or barometer of heart failure severity, but can also be a contributor to heart failure itself. In the present study, the AF-FTR patients shared clinical characteristics with patients defined as classical HFpEF condition. The AF-FTR patients consisted of elderly patients with typical comorbidities seen in AF populations such as hypertension, diabetes mellitus in accordance with preserved LVEF and no signs of valve disease. The AF-FTR patients were highly symptomatic with significantly increased NT-pro-BNP despite all patients were preload optimized with diuretics and had stable rate-controlled AF. The exercise capacity was severely impaired with an average peak consumption of 15 ml/kg/min which was comparable to the CA group. The low exercise capacity could be due to an inadequate chronotropic response following treatment with betablockers. However, this seems unlikely as the average peak heart rate during exercise reached 131 beats per minute accounting for 80–85% of the estimated maximal heart rate. The exercise test was performed in a semi-supine position which might have influenced the maximal exercise capacity achieved but the serum lactate was above 5 mmol/l at peak indicating that the patients were under substantial strain. Overall, the exercise capacity and response in AF-FTR was comparable with patients with clinical heart failure due to restrictive amyloid cardiomyopathy with preserved LVEF.

At rest, CO was reduced in both patient groups with a significantly lower CO in the AF-FTR patients which is in accordance with previous findings [27]. The CO reserve during exercise was impaired to similar degree in both groups with only a two-fold raise in CO. The impairment of resting and exercise CO is in accordance with a previous study in severe FTR but with some difference in baseline patient characteristics [28]. In addition, the ratio of ΔVO2/ΔCO was severely reduced as 1 ml/min increase in VO2 was only followed by an approximately 3 ml/min increase in CO which is opposed by 1 ml/min VO2 to 6 ml/min CO relationship in normal subjects [29].

Due to the regurgitation volume among the AF-FTR patients, the forward output is reduced. Therefore, CO assessed by Fick´s principle during the RHC demonstrated reduced CO at rest and at peak exercise in AF-FTR even though the RV myocardial performance assessed by 2D- and 3D echocardiography demonstrated normal values of RVEF, TAPSE and S´. However, the echocardiographic RV systolic parameters might be overestimating the systolic myocardial performance due to a reduced RV afterload as a consequence of a significant tricuspid regurgitant volume. Based on the RVSWI (lower normal: 8 gm-m/m^2^/beat) and the CO data some degree of impaired RV performance seems to be present due to the TR itself as well as the coexisting increased pulmonary venous pressure that might reduce the RV compliance and contribute to an abnormal RV performance in AF-FTR patients. It is also noteworthy that the RVSWI did not differ from the control patients representing an infiltrative restrictive cardiomyopathy entity that usually also involves the RV to some extent.

Severe bi-atrial enlargement (in particular RA enlargement) was noted along with moderated elevated resting atrial filling pressures in AF-FTR patients which is in accordance with the findings reported by Borlaug et al. [28], and in patients with and without AF [5]. With exercise, the mean RAP raised significantly doubling the pressure and significantly exceeding the level of RAP found in the CA group. The PCWP at peak exercise increased further exceeding 30 mmHg in AF-FTR resembling the same degree of response seen in HFpEF patients [9, 30]. AF-FTR patients share some common clinical and hemodynamic features with the HFpEF population but differ from those in some aspects as the right and left filling pressures at rest are elevated and the CO is reduced at rest, findings that is not usually seen in HFpEF patients [9]. The LV preload was assessed by LVMTP which differed significantly between the AF-FTR and CA control group as the slope of the rest-to exercise relation curve of LVMTP and CO were shallower in CA patients in comparison to AF-FTR. This finding could be expected due to the well-known restrictive LV pathophysiology seen in amyloid cardiomyopathy patients. In contrast to the findings by Borlaug et al., we found a positive and steep significant relation between LVMTP and CO comparable to normal subjects indicating that the LV preload is less affected in AF-FTR patients. This is even though the left atrial contraction component is absent due to AF in the FTR patients. However, the increased PCWP at rest and at peak might to some degree compensate the absent atrial contraction component and thereby maintain the LV filling in AF-FTR. Subjects with AF-FTR displayed a minor increase in CO despite a greater increase in RAP than CA control patients. In contrast, the relation between the increase of CO to PCWP was nearly similar in the two patient groups.

In the past recent years different transcatheter technologies have emerged as alternative to conventional surgery to high-risk populations with symptomatic significant TR. Recommendations regarding interventions indications and choice of imaging pre-and during procedure has been suggested and enables to predict risk of therapeutic failure [31–33]. The growing clinical importance of secondary isolated TR contributing to heart failure symptoms and significant altered hemodynamics is supported by the present study. However, whether transcatheter TV interventions can improve the abnormal hemodynamics in AF-FTR and translate this into an improvement of symptoms, physical capacity and even prognosis need to be clarified by future investigations.

Some limitations have to be taken into consideration beyond the experience of a single center and the small cohort of patients. First, the AF-FTR patients studied were all patients that were able to perform a semi-supine bicycle test excluding patients with physical disabilities or even just the inability to perform a stress test which is often seen in this patient population with advanced age. However, the patients were selected from the out-patient clinic where they were referred for unexplained dyspnea, leg edema or cardiac murmur evaluation and not from a patient population referred for invasive hemodynamic evaluation or referred for surgery or percutaneous TR intervention. Second, we used a semi-supine bicycle test which might have reduced the true maximal objective workload, but heart rate achieved and serum lactate level indicates that the patients were stressed sufficiently. Third, the difference in age, diabetes, and hypertension between the two groups might have influenced our results. However, there was no difference in LVEF, levels of NT-proBNP and hemodynamics including PCWP between the two groups at rest and PCWP increased significantly to the same extent in both groups indicating that the differences did not severely influenced our results. Fourth, we did not measure frailty score or lung function in our populations, but all the patients were without pulmonary disease and able to use a semi-supine bicycle test which indicated a reasonable performance. There was no difference in the VO2 consumption and the mean pulmonary artery pressure at rest and during peak exercise indicating that there was no severe difference in the lung function influencing the hemodynamics between the groups. The CA group was chosen as a recognized heart failure population requiring diuretic treatment despite LVEF is in the normal range in order to enlighten how symptomatic the population of AF-FTR is.

## Conclusion

The present study supports the growing recognition that significant AF-FTR contributes to development of advanced heart failure symptoms, increased natriuretic peptides, and poor exercise capacity. Another feature is significantly altered hemodynamics with increased atrial filling pressures, reduced cardiac output at rest and during exercise and thereby sharing some common features with seen in HFpEF patients with other etiologies.

## Data Availability

The datasets generated and/or analysed during the current study are not publicly available due plans of additional publications later on from this dataset, but are available from the corresponding author on reasonable request.
